# A Retrospective Study of First‐Line Immunotherapy for Advanced Non‐Small Cell Lung Cancer in the Elderly

**DOI:** 10.1002/agm2.70052

**Published:** 2025-12-26

**Authors:** Sheng Yang, Yalin Zhu, Sitong Liu, Kui Xiao

**Affiliations:** ^1^ Department of Pulmonary and Critical Care Medicine, The Second Xiangya Hospital Central South University Changsha Hunan China; ^2^ Research Unit of Respiratory Disease Central South University Changsha Hunan China; ^3^ Clinical Medical Research Center for Pulmonary and Critical Care Medicine in Hunan Province Changsha Hunan China; ^4^ Diagnosis and Treatment Center of Respiratory Disease in Hunan Province Changsha Hunan China

**Keywords:** elderly, geriatric, immune checkpoint inhibitors, non‐small cell lung cancer, pembrolizumab, tislelizumab

## Abstract

**Objectives:**

In this study, we compared the efficacy and safety of first‐line IO among different age subgroups of elderly patients, as well as in comparison to non‐elderly patients with advanced NSCLC. Additionally, we investigated hematological biomarkers associated with overall survival (OS) and compared the efficacy and safety of pembrolizumab plus chemotherapy (CT) versus tislelizumab plus CT in the treatment of advanced NSCLC.

**Methods:**

We conducted a retrospective analysis of clinical data from 298 patients with stage IIIB–IVB NSCLC without driver mutations who received first‐line IO at The Second Xiangya Hospital of Central South University between June 1, 2019, and June 30, 2023. Differences between elderly and non‐elderly patients, as well as among different elderly age subgroups, were analyzed using the chi‐squared test and the Kruskal–Wallis *H* test. Receiver operating characteristic (ROC) curves were used to determine the optimal cut‐off values for hematological biomarkers. Patients were categorized into two groups based on treatment regimens: pembrolizumab plus CT and tislelizumab plus CT. To minimize baseline differences between the groups, 1:1 propensity score matching (PSM) was applied. A two‐sided *p*‐value of < 0.05 was considered statistically significant.

**Results:**

The study population was divided into two groups: elderly group (≥ 65 years old, *n* = 145 cases) and non‐elderly group (< 65 years old, *n* = 153 cases). The elderly group was further divided into three subgroups: 65–69 years old group (*n* = 78 cases), 70–74 years old group (*n* = 48 cases), and ≥ 75 years old group (*n* = 19 cases). Kaplan–Meier survival analysis using the log‐rank test revealed no statistically significant difference in OS among the age groups (*p* = 0.100). Multivariate COX regression analysis indicated that smoking history, bone metastasis, PD‐L1 tumor proportion score (TPS), and prognostic nutritional index (PNI) are independent prognostic factors for OS. Monocyte count (M), NLR, PLR, MLR, PIV, and SII were negatively correlated with OS, while PNI was positively associated with OS in patients with advanced NSCLC. Before PSM, the median OS (mOS) for patients receiving pembrolizumab plus CT and tislelizumab plus CT was 39.180 months (95% CI: 25.440–NA) and not reached (95% CI: 42.110–NA), respectively, with no statistically significant difference (*p* = 0.287). After PSM, the mOS was 46.150 months (95% CI: 17.550–NA) in the pembrolizumab plus CT group, while it remained not reached (95% CI: 20.550–NA) in the tislelizumab plus CT group, again show no statistically significant difference (*p* = 0.346).

**Conclusion:**

Age may not be the main prognostic factor for the effectiveness of first‐line immunotherapy in patients with advanced NSCLC. Longer mOS was observed in patients who were non‐smokers, had no bone metastases, exhibited PD‐L1 TPS ≥ 50%, and had a higher prognostic nutritional index (PNI), regardless of age. Furthermore, the efficacy and safety profiles of pembrolizumab plus CT versus tislelizumab plus CT show no differences in the treatment of advanced NSCLC.

## Graphical abstract

This is a retrospective study of the efficacy and safety of first‐line immunotherapy in elderly patients with advanced NSCLC. The hematologic prognostic markers of first‐line immunotherapy in advanced NSCLC patients, especially PNI, were investigated. This is the first retrospective study to evaluate the efficacy and safety of pembrolizumab plus CT versus tislelizumab plus CT in patients with advanced NSCLC.

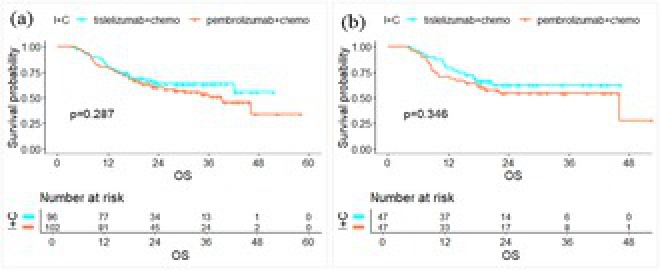

## 1 Introduction

According to global statistics in 2022, lung cancer is the tumor with the highest morbidity and mortality rate in the world, with approximately 2.48 million new cases and 1.87 million deaths, accounting for 12.4% and 18.7% of the tumors diagnosed worldwide respectively [1]. Lung cancer is classified based on its pathological characteristics into two main types: NSCLC and small cell lung cancer (SCLC). Among these, NSCLC is the more common type, accounting for about 85% of all lung cancer cases. NSCLC is mainly classified into adenocarcinoma, squamous carcinoma, and large cell carcinoma [2, 3]. Most patients are initially diagnosed with advanced lung cancer and are aged 65 years or older [4]. Despite this, most clinical trials included younger patients, with fewer elderly patients, especially those over 75 years old, and most of them were patients with fewer comorbidities and a performance status (PS) score of 0–1 [5, 6, 7, 8]. The main treatment modalities for advanced NSCLC include immune checkpoint inhibitors (ICIs), molecular targeted therapy, chemotherapy, and anti‐angiogenic therapy, etc. The advent of molecular targeted therapy and ICIs has revolutionized the treatment landscape for advanced NSCLC, significantly prolonged survival [9]. ICIs mainly include PD‐1 inhibitors, PD‐L1 inhibitors, CTLA‐4 inhibitors, TIM‐ inhibitors, LAG‐3 inhibitors, and TIGIT inhibitors, etc. The first three types of them are the most commonly used in clinical practice. PD‐1 inhibitors can enhance the function of effector T cells by inhibiting the interaction between PD‐1 and PD‐L1 and PD‐L2, thereby enhancing the anti‐tumor response. CTLA‐4 inhibitors can also enhance the anti‐tumor response by inhibiting CTLA‐4 from competing with CD28 for binding to B7‐1 and B7‐2, thereby promoting T cell activation [10]. Immunosenescence is the process of remodeling of immune function during aging and is associated with a reduced ability of the immune system to mount an effective immune response. Senescence is associated with the senescence‐associated secretory phenotype (SASP), a complex mechanism characterized by elevated serum levels of pro‐inflammatory cytokines, such as IL‐1, IL‐6, IL‐8, and TNF ‐ α. SASP has been associated with poor efficacy of immune checkpoint blockade, as cytokines associated with SASP favor tumor progression [5, 11, 12]. Currently, multiple phase III randomized controlled clinical trials (RCTs) have shown that first‐line ICIs treatment significantly prolonged both median progression‐free survival (mPFS) and mOS in patients with advanced NSCLC compared to CT alone. These findings have been incorporated into authoritative guidelines such as NCCN, ASCO, ESMO, and CSCO [13, 14, 15, 16, 17, 18, 19]. For instance, the Keynote‐189 and Keynote‐407 studies demonstrated that pembrolizumab plus CT significantly improved the objective response rate (ORR), mPFS, and mOS in advanced NSCLC patients compared with CT alone [20, 21, 22]. Similarly, the Rational‐304 and Rational‐305 studies demonstrated that tislelizumab plus CT significantly improved ORR and mPFS compared to CT in advanced NSCLC [23, 24]. However, not all advanced NSCLC patients can benefit from ICIs plus CT. In several large‐scale clinical studies, the ORR of the ICIs plus CT group ranged from approximately 47% to 64% [22, 23, 25, 26, 27]. PD‐L1 is currently recognized as a predictive biomarker for IO. However, due to its temporal and spatial specificity differences, there are still many limitations in the prediction of efficacy [28, 29, 30, 31]. Multiple retrospective studies have shown that pre‐treatment laboratory indicators such as NLR, PLR, PNI, and SII can serve as effective predictors of ICIs treatment efficacy. These markers were easy to obtain and very cost‐effective [32, 33, 34, 35, 36, 37, 38]. Although pembrolizumab plus CT and tislelizumab plus CT both improved the survival outcomes of patients with advanced NSCLC, there are currently no direct head‐to‐head comparisons of the efficacy and safety of these two drugs. Therefore, this retrospective study mainly aims to address three key objectives: (1) To investigate whether elderly patients with advanced NSCLC can obtain survival benefits from first‐line ICIs treatment; (2) To explore predictive markers for the efficacy of first‐line ICIs treatment in advanced NSCLC; (3)To compare the efficacy and safety of pembrolizumab plus CT and tislelizumab plus CT in the treatment of advanced NSCLC.

## 2 Information and Methods

### 2.1 Case Data Collection

The clinical data of patients with advanced NSCLC who received first‐line ICIs treatment in The Second Xiangya Hospital of Central South University from June 1, 2019, to June 30, 2023, were retrospectively collected. Data included the patients' age, gender, PS score, smoking history, comorbidities, pathological type, TNM stage, metastasis sites, PD‐L1 expression levels, ICI drugs and regimens, pre‐treatment laboratory results (neutrophils, lymphocytes, monocytes, eosinophils, basophils, albumin, etc.), and treatment‐related adverse events (TRAEs). All data were collected through the hospital's electronic medical record system. The study was approved by the Ethics Committee of The Second Xiangya Hospital of Central South University (LYF2024299) and was granted an exemption from obtaining informed consent.

### 2.2 Inclusion and Exclusion Criteria

*Inclusion criteria*: (1) Pathological diagnosis of NSCLC with clinical stage of IIIB‐IV according to the American Joint Committee on Cancer (AJCC) 8th edition of lung cancer staging criteria; (2) age ≥ 18 years; (3) negative for driver gene mutations such as EGFR, ALK, and ROS1; (4) Received ≥ two cycles of first‐line ICIs treatment; (5) availability of complete clinical data.

*Exclusion criteria*: (1) Concurrent with primary malignant tumors at other sites; (2) diagnosis of autoimmune diseases, primary/acquired immune deficiency disorders, or patients requiring long‐term corticosteroid and/or immunosuppressant therapy prior to ICIs treatment; (3) incomplete clinical data; and (4) received less than two cycles of ICIs treatment

### 2.3 Study Group

Based on the age distribution of the included patients, they were divided into the elderly group and the non‐elderly group. The age limit was set at 65 years. The elderly group was further subdivided into three subgroups: the 65–69 years group, the 70–74 years group, and the ≥ 75 years group. Patients were also divided into two treatment groups by ICI drug they received: pembrolizumab plus CT and tislelizumab plus CT.

### 2.4 Evaluation of the Efficacy

Imaging evaluation was based on Response Evaluation Criteria in Solid Tumors, version 1.1 (RECIST v1.1), which includes the categories: complete response (CR), partial response (PR), stable disease (SD), and progressive disease (PD). The primary endpoint of the study is OS, defined as the time from the administration of the first‐line ICIs treatment to either death or the end of the follow‐up period. Secondary endpoints included ORR as the ratio of the number of CR and PR to the total number of cases, and disease control rate (DCR) as the ratio of the number of CR, PR and SD to the total number of cases. The follow‐up date for the study was set to *October 15, 2024*, with events that did not reach the predefined endpoint treated as *censored data*. *TRAEs* were assessed based on data collected through the hospital's electronic medical record system.

### 2.5 Follow Up

Patients' survival information were obtained through records of outpatient follow‐up or inpatient treatment in the electronic medical record system of the Second Xiangya Hospital of Central South University, regular return visits, and telephone calls to the included population.

### 2.6 Statistical Analysis

For the clinical baseline characteristics of the included population, count data were expressed as frequencies and percentages, and the chi‐squared test or Fisher's exact probability method were used between groups comparison. Measurement data that satisfied normal distribution were expressed as the mean and standard error, with one‐way Analysis of Variance (ANOVA) used for comparisons between more than two groups and the *t*‐test for comparisons between two groups. For data that did not follow a normal distribution, values were expressed as the median and interquartile range, and the Kruskal–Wallis *H* test was used for comparisons between more than two groups, while the Mann–Whitney *U* test was used for comparisons between two groups. Predictors of OS were analyzed using univariate and multivariate COX regression. ROC curves were used to calculate cut‐off values for laboratory results and to analyze hematological predictive markers of OS. Propensity score matching (PSM) for nearest neighbor conditioned on baseline covariates (age group, gender, smoking history, pathology type, stage and PD‐L1 TPS) was performed on a 1:1 basis with a caliper width of 0.02. The Kaplan–Meier curve analysis was employed to examine the differences in mOS among different age groups and different ICIs drugs. All statistical analyses were performed by SPSS (version 27.0) and R (version 4.4.1) statistical software.

## 3 Results

### 3.1 Baseline Characteristics

A total of 298 patients with advanced NSCLC were included in this study. The demographic characteristics of the patients are summarized in Table 1. The median age was 64 years, with a range from 36 to 85 years. The distribution of patients across age groups was as follows: 153 patients (51.3%) in the < 65 years old group, 78 patients (26.2%) in the 65–69 years old group, 48 patients (16.1%) in the 70–74 years old group, and 19 patients (6.4%) in the ≥ 75 years old group. Statistically significant differences were observed in the prevalence of chronic obstructive pulmonary disease (COPD), hypertension, and the number of patients receiving radiotherapy across different age groups (*p* < 0.05). Of the enrolled patients, 91.6% were male and 8.4% were female. Regarding histological classification, squamous cell carcinoma accounted for 59.7%, while adenocarcinoma made up 38.3%. There were 156 patients (52.3%) in stage IV NSCLC, among whom 74 had bone metastasis, and 44.3% were in stage IIIB–IIIC. In terms of PD‐L1 expression, 16.1% of patients had < 1% expression, 42.6% had 1%–49% expression, and 41.3% had ≥ 50% expression. With regard to treatment regimen, 266 patients (89.3%) received a combination of ICIs plus chemotherapy. Other treatment regimens included ICIs monotherapy (6.7%), ICIs plus anti‐angiogenic therapy (3.0%), and ICIs combined with both CT and anti‐angiogenic therapy (1.0%). Pembrolizumab and tislelizumab were the most commonly used ICIs, administered to 124 (41.6%) and 103 (34.6%) patients, respectively. Other ICIs used included sintilimab (16.4%), camrelizumab (4.0%), toripalimab (2.3%), sugemalimab (0.7%), and nivolumab (0.3%). Among the laboratory indicators, the monocyte‐to‐lymphocyte ratio (MLR) and prognostic nutritional index (PNI) showed statistically significant differences across the various age groups (*p* < 0.05).

**TABLE 1:** The demographic and clinical characteristics of 298 NSCLC patients who received first‐line ICIs treatment.

Characteristics	Overall (*n* = 298)	< 65 (*n* = 153)	65–69 (*n* = 78)	70–74 (*n* = 48)	≥ 75 (*n* = 19)	*p*
Age	64.00[58.00,69.00]	58.00[54.00,62.00]	67.00[66.00,68.00]	72.00[70.00,73.00]	79.00[77.00,79.50]	< 0.001*
Sex						0.947
Male	273 (91.6)	140 (91.5)	72 (92.3)	43 (89.6)	18 (94.7)	
Female	25 (8.4)	13 (8.5)	6 (7.7)	5 (10.4)	1 (5.3)	
Smoking						0.786
No	65 (21.9)	31 (20.4)	19 (24.4)	12 (25.0)	3 (15.8)	
Yes	232 (78.1)	121 (79.6)	59 (75.6)	36 (75.0)	16 (84.2)	
COPD						0.011*
Yes	34 (11.4)	11 (7.2)	8 (10.3)	10 (20.8)	5 (26.3)	
No	264 (88.6)	142 (92.8)	70 (89.7)	38 (79.2)	14 (73.7)	
HBP						< 0.001*
Yes	69 (23.2)	21 (13.7)	23 (29.5)	16 (33.3)	9 (47.4)	
No	229 (76.8)	132 (86.3)	55 (70.5)	32 (66.7)	10 (52.6)	
Diabetes						0.076
Yes	34 (11.4)	12 (7.8)	11 (14.1)	6 (12.5)	5 (26.3)	
No	264 (88.6)	1 (92.2)	67 (85.9)	42 (87.5)	14 (73.7)	
Hepatitis B						0.937
Yes	18 (6.0)	11 (7.2)	4 (5.1)	2 (4.2)	1 (5.3)	
No	280 (94.0)	142 (92.8)	74 (94.9)	46 (95.8)	18 (94.7)	
CVD						0.070
Yes	21 (7.0)	8 (5.2)	4 (5.1)	8 (16.7)	1 (5.3)	
No	277 (93.0)	145 (94.8)	74 (94.9)	40 (83.3)	18 (94.7)	
Obstructive pneumonia						0.511
Yes	44 (14.8)	26 (17.0)	12 (15.4)	5 (10.4)	1 (5.3)	
No	254 (85.2)	127 (83.0)	66 (84.6)	43 (89.6)	18 (94.7)	
Pathology						0.698
Adenocarcinoma	114 (38.3)	59 (38.6)	28 (35.9)	17 (35.4)	10 (52.6)	
Squamous	178 (59.7)	92 (60.1)	47 (60.3)	30 (62.5)	9 (47.4)	
Others	6 (2.0)	2 (1.3)	3 (3.8)	1 (2.1)	0 (0.0)	
Stage						0.282
IIIB/C	132 (44.3)	67 (43.8)	35 (44.9)	21 (43.8)	9 (47.4)	
IV	156 (52.3)	79 (51.6)	43 (55.1)	26 (54.2)	8 (42.1)	
NA	10 (3.4)	7 (4.6)	0 (0.0)	1 (2.1)	2 (10.5)	
Liver						0.859
Yes	15 (5.0)	8 (5.2)	4 (5.1)	3 (6.2)	0 (0.0)	
No	283 (95.0)	145 (94.8)	74 (94.9)	45 (93.8)	19 (100.0)	
Bone						0.970
Yes	74 (24.8)	38 (24.8)	19 (24.4)	13 (27.1)	4 (21.1)	
No	224 (75.2)	115 (75.2)	59 (75.6)	35 (72.9)	15 (78.9)	
Brain						0.529
Yes	28 (9.4)	14 (9.2)	9 (11.5)	5 (10.4)	0 (0.0)	
No	270 (90.6)	139 (90.8)	69 (88.5)	43 (89.6)	19 (100.0)	
Plerua						0.560
Yes	33 (11.1)	14 (9.2)	11 (14.1)	5 (10.4)	3 (15.8)	
No	265 (88.9)	139 (90.8)	67 (85.9)	43 (89.6)	16 (84.2)	
Lung						0.484
Yes	40 (13.4)	24 (15.7)	11 (14.1)	4 (8.3)	1 (5.3)	
No	258 (86.6)	129 (84.3)	67 (85.9)	44 (91.7)	18 (94.7)	
PD‐L1 (TPS)						0.760
< 1%	48 (16.1)	24 (15.7)	14 (17.9)	5 (10.4)	5 (26.3)	
1%–49%	127 (42.6)	68 (44.4)	32 (41.0)	20 (41.7)	7 (36.8)	
≥ 50%	123 (41.3)	61 (39.9)	32 (41.0)	23 (47.9)	7 (36.8)	
Therapy						0.127
I	20 (6.7)	8 (5.2)	5 (6.4)	4 (8.3)	3 (15.8)	
I + C	266 (89.3)	141 (92.2)	71 (91.0)	40 (83.3)	14 (73.7)	
I + A	9 (3.0)	3 (2.0)	1 (1.3)	3 (6.2)	2 (10.5)	
I + C + A	3 (1.0)	1 (0.7)	1 (1.3)	1 (2.1)	0 (0.0)	
Immunotherapy						NA
Pembrolizumab	124 (41.6)	63 (41.2)	31 (39.7)	14 (29.2)	16 (84.2)	
Tislelizumab	103 (34.6)	45 (29.4)	33 (42.3)	23 (47.9)	2 (10.5)	
Sintilimab	49 (16.4)	33 (21.6)	8 (10.3)	7 (14.6)	1 (5.3)	
Camrelizumab	12 (4.0)	6 (3.9)	4 (5.1)	2 (4.2)	0 (0.0)	
Torpalimab	7 (2.3)	4 (2.6)	1 (1.3)	2 (4.2)	0 (0.0)	
Sugemalimab	2 (0.7)	1 (0.7)	1 (1.3)	0 (0.0)	0 (0.0)	
Nivolumab	1 (0.3)	1 (0.7)	0 (0.0)	0 (0.0)	0 (0.0)	
Radiotherapy						0.004*
Yes	61 (20.5)	41 (26.8)	16 (20.5)	3 (6.2)	1 (5.3)	
No	237 (79.5)	112 (73.2)	62 (79.5)	45 (93.8)	18 (94.7)	
M (×10^9^/L)						0.251
< 0.385	98 (32.9)	58 (37.9)	23 (29.5)	13 (27.1)	4 (21.1)	
≥ 0.385	200 (67.1)	95 (62.1)	55 (70.5)	35 (72.9)	15 (78.9)	
NLR						0.244
< 4.278	199 (66.8)	106 (69.3)	51 (65.4)	27 (56.2)	15 (78.9)	
≥ 4.278	99 (33.2)	47 (30.7)	27 (34.6)	21 (43.8)	4 (21.1)	
PLR						0.023*
< 168.88	132 (44.3)	76 (49.7)	23 (29.5)	23 (47.9)	10 (52.6)	
≥ 168.88	166 (55.7)	77 (50.3)	55 (70.5)	25 (52.1)	9 (47.4)	
MLR						0.244
< 0.298	199 (66.8)	106 (69.3)	51 (65.4)	27 (56.2)	15 (78.9)	
≥ 0.298	99 (33.2)	47 (30.7)	27 (34.6)	21 (43.8)	4 (21.1)	
SII						0.496
< 707.017	99 (33.2)	57 (37.3)	22 (28.2)	14 (29.2)	6 (31.6)	
≥ 707.017	199 (66.8)	96 (62.7)	56 (71.8)	34 (70.8)	13 (68.4)	
PIV						0.499
< 207.997	62 (20.8)	37 (24.2)	13 (16.7)	8 (16.7)	4 (21.1)	
≥ 207.997	236 (79.2)	116 (75.8)	65 (83.3)	40 (83.3)	15 (78.9)	
PNI						0.003*
< 44.025	84 (28.2)	30 (19.6)	28 (35.9)	21 (43.8)	5 (26.3)	
≥ 44.025	214 (71.8)	123 (80.4)	50 (64.1)	27 (56.2)	14 (73.7)	

*Note:* PIV = neutrophil × monocyte × PLT/lymphocyte, PNI = ALB (g/L) + 5 × lymphocyte.

Abbreviations: A, antiangiogenic therapy; Baso, basophil; C, chemotherapy; COPD, chronic obstructive pulmonary disease; CVD, coronary heart disease; E, eosinophils; I, immunotherapy; L, lymphocyte; M, monocyte; MLR, monocyte/lymphocyte; N, neutrophil, NLR, N/lymphocyte, PLR, PLT/lymphocyte, PIV Pan‐Immune‐Inflammation Value, PNI Prognostic Nutritional Index, MLR Monocyte‐to‐Lymphocyte Ratio, NLR Neutrophil‐to‐Lymphocyte Ratio, PLR Platelet‐to‐Lymphocyte Ratio.

*p < 0.05, the difference is statistically significant.

### 3.2 Radiological Evaluation

In the enrolled population, the efficacy of each two‐cycle treatment with ICIs ± CT± anti‐angiogenic therapy was evaluated based on the imaging examination results. Among these patients, 1 achieved CR, 138 obtained PR, 142 had SD, and 17 experienced PD. When stratified by age, the ORR was 36.6% in patients aged < 65 years old group, 59.0% in those aged 65–69 years old group, 60.4% in the 70–74 years old group, and 42.1% in patients aged ≥ 75 years old group, with a statistically significant difference among groups (*p* = 0.002). Pairwise comparisons showed no significant difference in ORR between the 70–74 years old group and the ≥ 75 years or 65–69 years old groups; however, all three older groups had a significantly higher ORR compared to the < 65 years old group. No significant differences in DCR were observed among the age groups (*p* = 0.078) (Table 2).

**TABLE 2:** Radiological evaluation of the included population.

Radiological evalution	Overall	< 65	65–69	70–74	≥ 75	*p*
CR	1 (0.3)	0 (0.0)	1 (1.3)	0 (0.0)	0 (0.0)	
PR	138 (46.3)	56 (36.6)	45 (57.7)	29 (60.4)	8 (42.1)	
SD	142 (47.7)	86 (56.2)	30 (38.5)	18 (37.5)	8 (42.1)	
PD	17 (5.7)	11 (7.2)	2 (2.6)	1 (2.1)	3 (15.8)	
ORR (%)	46.6	36.6	59	60.4	32.1	0.002*
DCR (%)	94.3	92.8	97.4	97.9	84.2	0.078

* *p* < 0.05, the difference is statistically significant.

### 3.3 Kaplan–Meier Curves Stratified by Age and PD‐L1 Subgroups

**FIGURE 1:**
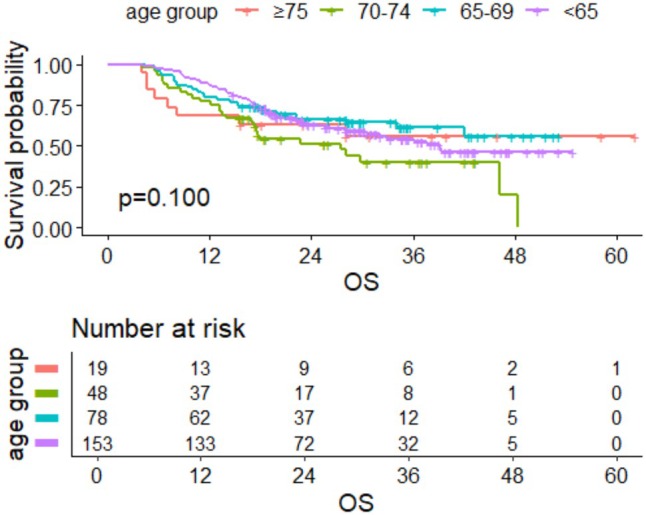
Survival curves of OS for the four age subgroups in the total population.

**FIGURE 2:**
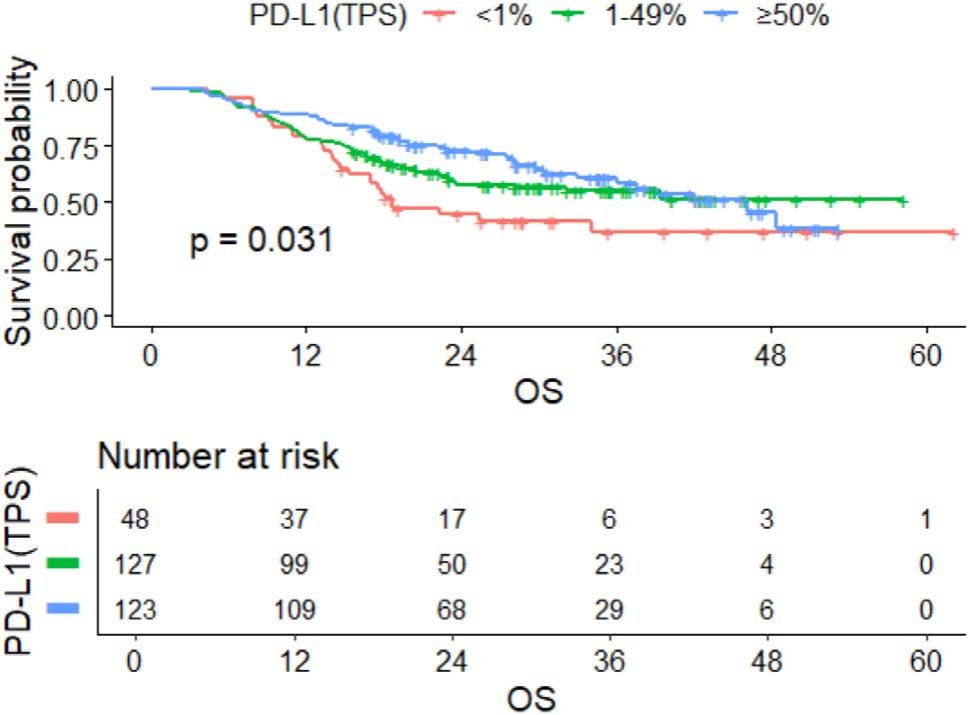
Kaplan–Meier curve of OS in the total population grouped by PD‐L1 (TPS).

By the last follow‐up time, a total of 129 individuals died among the included population. Among them, 66 deaths occurred in the < 65 years old group, 28 in the 65–69 years old group, 27 in the 70–74 years old group, and 8 in the ≥ 75 years old group. Kaplan–Meier(K‐M) survival analysis showed that mOS for the entire cohort was 39.350 months (95% CI: 32.050–NA). Based on the log‐rank test, the mOS of the < 65 years old group, 65–69 years old group, 70–74 years old group, and ≥ 75 years old group were 39.180 months (95% CI: 30.570–NA), NA (95% CI: 42.110–NA), 27.550 months (95% CI: 17.190–NA), and NA (95% CI: 15.450–NA), respectively (Figure 1). Through the log‐rank test, there was no significant difference in mOS among the four age groups (*p* = 0.100). When stratified by PD‐L1 tumor proportion score (TPS) levels (< 1%, 1%–49%, and ≥ 50%), significant differences in mOS were observed among the three groups (*p* = 0.031), with the PD‐L1 ≥ 50% group exhibiting the longest mOS at 46.150 months (Figure 2).

### 3.4 Predictive Markers of OS

**FIGURE 3:**
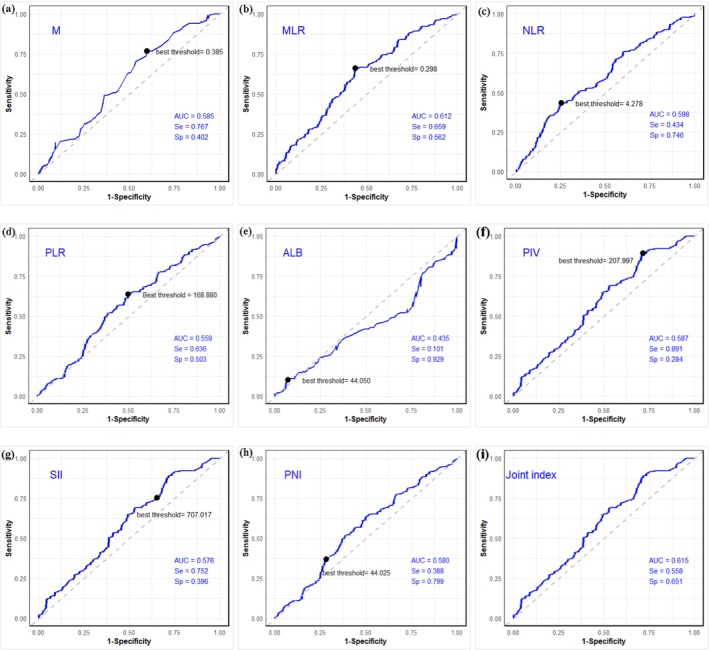
ROC curves of hematological indicators: (a) Monocyte (M); (b) MLR; (c) NLR; (d) PLR; (e) albumin (ALB); (f) PIV; (g) SII; (h) PNI; and (i) joint index. AUC, area under the curve; Se, sensitivity; Sp, specificity.

**FIGURE 4:**
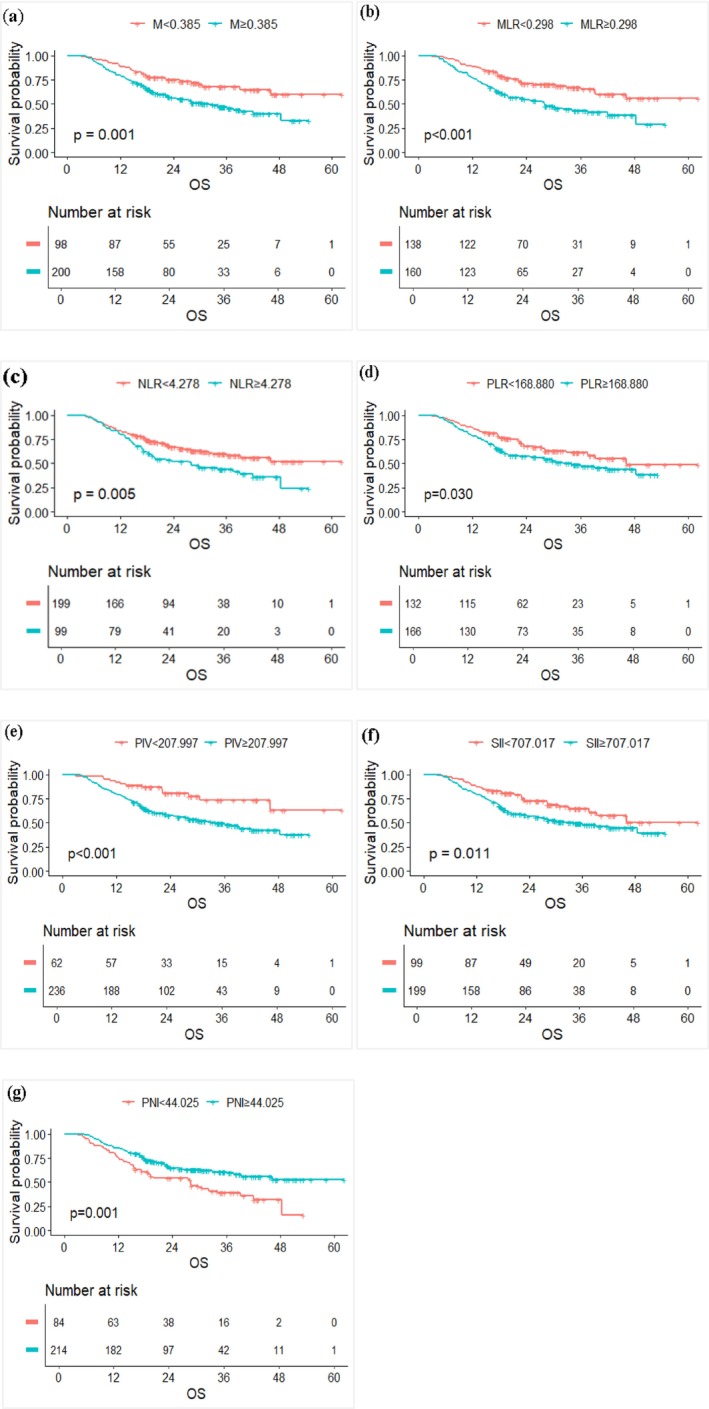
OS survival curves stratified by hematological indicators: (a) Monocyte (M); (b) MLR; (c) NLR; (d) PLR; (e) PIV; (f) SII; and (g) PNI.

Based on survival status, the cutoff values of M, NLR, MLR, PLR, SII, PIV, PNI, and ALB were calculated through the ROC curve as 0.385, 4.278, 0.298, 168.880, 707.017, 207.997, 44.025, and 44.050, respectively. The corresponding AUC values were 0.585, 0.598, 0.612, 0.559, 0.576, 0.587, 0.580, and 0.435, respectively, indicating that M, NLR, MLR, PLR, SII, PIV, and PNI may be associated with the efficacy of first‐line ICIs treatment in patients with advanced NSCLC, while ALB was not (Figures 3 and 4). When combining all laboratory indicators except ALB, the AUC increased to 0.615. Univariate regression analysis for OS showed that M, NLR, MLR, PLR, SII, PIV, and PNI were significant predictors of OS. Further multivariate regression analysis identified PNI as an independent predictor of OS (Table 3). In conclusion, M, NLR, MLR, PLR, SII, PIV, PNI, and SII (especially PNI) serve as predictive markers for OS in patients with advanced NSCLC receiving first‐line ICIs therapy.

**TABLE 3:** The univariate COX regression and multivariate COX regression of OS.

Factors	Univariate analysis		Multivariate analysis	
	HR (95% CI)	*p*	HR (95% CI)	*p*
Sex				
Male				
Female	0.782 (0.397–1.540)	0.477		
Age				
< 65				
65–69	0.870 (0.559–1.353)	0.536	0.806 (0.507–1.282)	0.364
70–74	1.620 (1.034–2.536)	0.035*	1.554 (0.949–2.546)	0.080
≥ 75	1.087 (0.521–2.267)	0.825	1.075 (0.497–2.325)	0.854
Smoking				
No				
Yes	1.758 (1.099–2.813)	0.019*	1.660 (1.032–2.671)	0.037*
COPD				
No				
Yes	1.284 (0.780–2.113)	0.326		
HBP				
No				
Yes	0.995 (0.658–1.505)	0.981		
Hepatitis B				
No				
Yes	0.777 (0.362–1.665)	0.516		
Diabetes				
No				
Yes	1.348 (0.809–2.246)	0.252		
CVD				
No				
Yes	0.819 (0.400–1.675)	0.584		
Obstructive pneumonia				
No				
Yes	0.849 (0.509–1.415)	0.529		
Pathology				
Adenocarcinoma				
Squamous carcinoma	0.944 (0.661–1.349)	0.752		
Others	2.915 (0.908–9.364)	0.072		
Stage				
IIIB/C				
IV	1.759 (1.214–2.549)	0.003*	1.312 (0.834–2.063)	0.239
NA	1.884 (0.802–4.430)	0.146	1.788 (0.727–4.393)	0.205
Liver metastasis				
No				
Yes	1.032 (0.482–2.212)	0.935		
Bone metastasis				
No				
Yes	2.007 (1.392–2.893)	< 0.001*	2.008 (1.276–3.159)	0.003*
Brain metastasis				
No				
Yes	1.094 (0.617–1.941)	0.758		
Plerua metastasis				
No				
Yes	1.461 (0.897–2.380)	0.128		
Lung metastasis				
No				
Yes	1.338 (0.831–2.156)	0.231		
Pericardium metastasis				
No				
Yes	2.417 (0.766–7.623)	0.132		
PD‐L1 (TPS)				
< 1%				
1%–49%	0.665 (0.421–1.052)	0.084	0.694 (0.440–1.094)	0.116
≥ 50%	0.537 (0.336–0.857)	0.004*	0.447 (0.275–0.726)	0.001*
Therapy				
ICIs + C				
ICIs	1.055 (0.566–1.966)	0.867		
ICIs + A	1.478 (0.649–3.368)	0.353		
ICIs + C + A	1.823 (0.450–7.393)	0.400		
Cycle	0.957 (0.899–1.019)	0.174		
ICIs				
Pembrolizumab				
Tislelizumab	0.831 (0.555–1.245)	0.370		
Sintilimab	0.716 (0.423–1.213)	0.214		
Camrelizumab	1.027 (0.444–2.378)	0.950		
Toripalimab	1.133 (0.451–2.845)	0.789		
Sugemalimab	1.540 (0.212–11.163)	0.669		
Nivolumab	Inf	0.995		
Radiotherapy				
No				
Yes	0.672 (0.424–1.064)	0.090		
M				
< 0.385				
≥ 0.385	1.965 (1.304–2.961)	0.001*	1.345 (0.819–2.208)	0.242
NLR				
< 4.278				
≥ 4.278	1.640 (1.157–2.324)	0.005*	0.901 (0.558–1.453)	0.668
MLR				
< 0.298				
≥ 0.298	2.005 (1.392–2.888)	< 0.001*	1.372 (0.799–2.355)	0.252
PLR				
< 168.880				
≥ 168.880	1.484 (1.036–2.125)	0.031*	1.135 (0.647–1.992)	0.659
PIV				
< 207.997				
≥ 207.997	2.578 (1.479–4.491)	0.001*	1.897 (0.912–3.945)	0.086
SII				
< 707.017				
≥ 707.017	1.670 (1.120–2.491)	0.012*	0.835 (0.433–1.608)	0.589
PNI				
< 44.025				
≥ 44.025	0.558 (0.391–0.796)	0.001*	0.601 (0.383–0.944)	0.027*
ALB				
< 44.05				
≥ 44.05	1.322 (0.745–2.3348)	0.340		
TRAE				
No				
Yes	0.900 (0.637–1.272)	0.552		

*Note:* *p < 0.05, the difference is statistically significant.

### 3.5 Base Univariate and Multivariate COX Regression of OS

Univariate Cox regression analysis for OS revealed that several factors were negatively associated with OS, including age 70–74 years, smoking history, clinical stage, presence of bone metastasis, monocyte count, MLR, NLR, PLR, PIV, and SII. In contrast, patients with PNI ≥ 44.025 and those with PD‐L1 TPS ≥ 50% demonstrated a better prognosis (Table 3). Compared with the < 65 years old group, the hazard ratios (HRs) for the 65–69 years and ≥ 75 years old groups were 0.87 and 1.09, respectively, with no significant differences observed (*p* > 0.05). However, patients aged 70–74 years old group had a 1.62‐fold increased risk of death compared to the < 65 years old group, reaching statistical significance (*p* = 0.035). Multivariate Cox regression analysis, conducted on variables with *p* < 0.05 in univariate analysis, identified smoking history, presence of bone metastasis, PNI, and PD‐L1 (TPS) as independent predictors of OS. Specifically, patients with smoking history and bone metastases had significantly poorer survival outcomes. Patients with a PD‐L1 TPS ≥ 50% had a 48% lower risk of death compared to those with PD ‐ L1 < 1% tumors (HR = 0.52, 95% CI: 0.32–0.85), which showed statistically significant (*p* = 0.009).

### 3.6 Comparison of the Efficacy of Pembrolizumab Plus CT and Tislelizumab Plus CT

**FIGURE 5:**
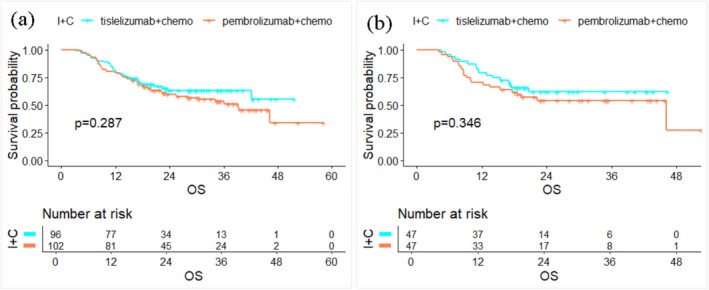
The OS survival curves of pembrolizumab + chemotherapy and tislelizumab + chemotherapy: (a) OS survival curves of the two groups of drugs before PSM and (b) OS survival curves of the two groups of drugs after PSM. I + C, immune checkpoint inhibitor combined with chemotherapy; pembrolizumab + chemo, pembrolizumab + chemotherapy; tislelizumab + chemo, tislelizumab + chemotherapy.

In this study, pembrolizumab and tislelizumab were the most commonly used first‐line ICIs for patients with advanced NSCLC. To further investigate efficacy differences, outcomes between patients treated with pembrolizumab plus CT and those treated with tislelizumab plus CT were compared. There were significant differences in smoking history, pathological type between the two groups. After 1:1 nearest‐neighbor PSM, a total of 94 patients were successfully matched, with a median age of 64 years old. By PSM, baseline characteristics between the two groups were well balanced (Table 4). Before PSM, the ORR of the pembrolizumab plus CT group and the tislelizumab plus CT group in the two groups was 46.1% and 54.2%, respectively, and the DCR was 95.1% and 95.8%, respectively. There were no statistically significant differences in ORR or DCR between the two groups before PSM. After PSM, the ORR and DCR remained comparable, with no significant differences observed (Table 5). Before PSM (*n* = 198), the mOS of the population was 42.110 months (95% CI: 36.090–NA). K–M survival analysis showed that the mOS for the pembrolizumab plus CT group was 39.180 months (95% CI:25.440‐NA), and the mOS of the tislelizumab plus CT was NA (95% CI: 42.110–NA), with no statistically significant difference (*p* = 0.287). After PSM (*n* = 94), the mOS of the total population was 46.150 months (95% CI: 22.160–NA). K–M analysis demonstrated that the mOS for the pembrolizumab plus CT group was 46.150 months (95% CI: 17.550–NA), and for the tislelizumab plus CT group, it was (95% CI: 20.550–NA), again with no statistically significant difference (*p* = 0.346). Overall, the efficacy of pembrolizumab plus CT and tislelizumab plus CT showed no differences in the treatment of advanced NSCLC (Figure 5).

**TABLE 4:** Baseline characteristics of the total population treated with pembrolizumab or tislelizumab (before and after PSM).

Variables	Before PSM				After PSM			
	Overall (*n* = 198)	Pembrolizumab + chemotherapy (*n* = 102)	Tislelizumab + chemotherapy (*n* = 96)	*p*	Overall (*n* = 94)	Pembrolizumab + chemotherapy (*n* = 47)	Tislelizumab + chemotherapy (*n* = 47)	*p*
Age	65.00 [58.00,69.00]	63.50 [57.00, 68.00]	65.00 [59.00, 69.00]	0.191	64.00 [58.00, 69.00]	64.00 [58.50, 69.00]	64.00 [55.00, 68.00]	0.261
Group				0.213				0.530
< 65	97 (49.0)	56 (54.9)	41 (42.7)		51 (54.3)	25 (53.2)	26 (55.3)	
65–69	57 (28.8)	25 (24.5)	32 (33.3)		25 (26.6)	11 (23.4)	14 (29.8)	
≥ 70	44 (22.2)	21 (20.6)	23 (24.0)		18 (19.1)	11 (23.4)	7 (14.9)	
Sex				0.073				1.000
Male	179 (90.4)	88 (86.3)	91 (94.8)		89 (94.7)	45 (95.7)	44 (93.6)	
Female	19 (9.6)	14 (13.7)	5 (5.2)		5 (5.3)	2 (4.3)	3 (6.4)	
Smoking				0.025*				1.000
Yes	150 (75.8)	70 (68.6)	80 (83.3)		75 (79.8)	37 (78.7)	38 (80.9)	
No	48 (24.2)	32 (31.4)	16 (16.7)		19 (20.2)	10 (21.3)	9 (19.1)	
COPD				1.000				0.111
Yes	22 (11.1)	11 (10.8)	11 (11.5)		7 (7.4)	6 (12.8)	1 (2.1)	
No	176 (88.9)	91 (89.2)	85 (88.5)		87 (92.6)	41 (87.2)	46 (97.9)	
HBP				0.457				1.000
Yes	49 (24.7)	28 (27.5)	21 (21.9)		20 (21.3)	10 (21.3)	10 (21.3)	
No	149 (75.3)	74 (72.5)	75 (78.1)		74 (78.7)	37 (78.7)	37 (78.7)	
Diabetes				0.327				0.316
Yes	22 (11.1)	14 (13.7)	8 (8.3)		10 (10.6)	7 (14.9)	3 (6.4)	
No	176 (88.9)	88 (86.3)	88 (91.7)		84 (89.4)	40 (85.1)	44 (93.6)	
Hepatitis B				0.499	4 (4.3)	4 (8.5)	0 (0.0)	0.117
Yes	9 (4.5)	6 (5.9)	3 (3.1)		90 (95.7)	43 (91.5)	47 (100.0)	
No	189 (95.5)	96 (94.1)	93 (96.9)					0.714
CVD				1.000	8 (8.5)	5 (10.6)	3 (6.4)	
Yes	19 (9.6)	10 (9.8)	9 (9.4)		86 (91.5)	42 (89.4)	44 (93.6)	
No	179 (90.4)	92 (90.2)	87 (90.6)					1.000
Pneumonia				0.233	11 (11.7)	5 (10.6)	6 (12.8)	
Yes	28 (14.1)	11 (10.8)	17 (17.7)		83 (88.3)	42 (89.4)	41 (87.2)	
No	170 (85.9)	91 (89.2)	79 (82.3)					1.000
Pathology				< 0.001	12 (12.8)	6 (12.8)	6 (12.8)	
Adenocarcinoma	57 (28.8)	50 (49.0)	7 (7.3)		80 (85.1)	40 (85.1)	40 (85.1)	
Squamous	136 (68.7)	48 (47.1)	88 (91.7)		2 (2.1)	1 (2.1)	1 (2.1)	
Others	5 (2.5)	4 (3.9)	1 (1.0)					0.534
Stage				0.365	51 (54.3)	27 (57.4)	24 (51.1)	
III	93 (47.0)	43 (42.2)	50 (52.1)		42 (44.7)	19 (40.4)	23 (48.9)	
IV	99 (50.0)	55 (53.9)	44 (45.8)		1 (1.1)	1 (2.1)	0 (0.0)	
NA	6 (3.0)	4 (3.9)	2 (2.1)					1.000
Liver metastasis				0.850	5 (5.3)	2 (4.3)	3 (6.4)	
Yes	12 (6.1)	7 (6.9)	5 (5.2)		89 (94.7)	45 (95.7)	44 (93.6)	
No	186 (93.9)	95 (93.1)	91 (94.8)		4 (4.3)	4 (8.5)	0 (0.0)	
Bone				0.332				1.000
Yes	44 (22.2)	26 (25.5)	18 (18.8)		18 (19.1)	9 (19.1)	9 (19.1)	
No	154 (77.8)	76 (74.5)	78 (81.2)		76 (80.9)	38 (80.9)	38 (80.9)	
Brain				0.893				1.000
Yes	16 (8.1)	9 (8.8)	7 (7.3)		7 (7.4)	4 (8.5)	3 (6.4)	
No	182 (91.9)	93 (91.2)	89 (92.7)		87 (92.6)	43 (91.5)	44 (93.6)	
Plerua				0.327				0.435
Yes	22 (11.1)	14 (13.7)	8 (8.3)		7 (7.4)	2 (4.3)	5 (10.6)	
No	176 (88.9)	88 (86.3)	88 (91.7)		87 (92.6)	45 (95.7)	42 (89.4)	
Lung				1.000				0.198
Yes	25 (12.6)	13 (12.7)	12 (12.5)		11 (11.7)	3 (6.4)	8 (17.0)	
No	173 (87.4)	89 (87.3)	84 (87.5)		83 (88.3)	44 (93.6)	39 (83.0)	
Radiotherapy				0.776				0.813
Yes	44 (22.2)	24 (23.5)	20 (20.8)		24 (25.5)	11 (23.4)	13 (27.7)	
No	154 (77.8)	78 (76.5)	76 (79.2)		70 (74.5)	36 (76.6)	34 (72.3)	
PD‐L1 (TPS)				0.193				0.911
< 1%	30 (15.2)	17 (16.7)	13 (13.5)		15 (16.0)	7 (14.9)	8 (17.0)	
1%–49%	88 (44.4)	39 (38.2)	49 (51.0)		35 (37.2)	17 (36.2)	18 (38.3)	
≥ 50%	80 (40.4)	46 (45.1)	34 (35.4)		44 (46.8)	23 (48.9)	21 (44.7)	
Cycle	4.00 [4.00, 4.00]	4.00 [4.00, 5.00]	4.00 [4.00, 4.00]	0.103	4.00 [4.00, 4.00]	4.00 [4.00, 5.00]	4.00 [4.00, 4.00]	0.343

*Note:* *p < 0.05, the difference is statistically significant.

**TABLE 5:** Radiological evaluations after treatment in the pembrolizumab group and the tislelizumab group (before and after PSM).

Radiological evalution	Before PSM (*n* = 198)			After PSM (*n* = 94)		
	Pembrolizumab + chemotherapy (*n* = 102)	Tislelizumab + chemotherapy (*n* = 96)	*p*	Pembrolizumab + chemotherapy (*n* = 47)	Tislelizumab + chemotherapy (*n* = 47)	*p*
CR	0	0	0	0	0	0
PR	47 (46.1)	52 (54.2)		22 (46.8)	25 (53.2)	
SD	50 (49.0)	40 (41.7)		23 (48.9)	20 (42.6)	
PD	5 (4.9)	4 (4.2)		2 (4.3)	2 (4.3)	
ORR (%)	46.1	54.2	0.255	46.8	53.2	0.680
DCR (%)	95.1	95.8	1.000	95.7	95.7	1.000

### 3.7 Safety Profiles

A total of 158 patients (53%) experienced TRAEs. The most common TRAEs were myelosuppression (28.2%), peripheral neuropathy (8.7%), and rash (7.7%). The incidence of TRAEs in the groups < 65 years old, 65–69 years old, 70–74 years old, and ≥ 75 years old was 46.4%, 60.3%, 60.4%, and 57.9%, respectively. There was no significant statistical difference in the probability of TRAE among all age groups (*p* = 0.135) (Table 6). Before PSM, the incidence of TRAEs was 45.1% in the pembrolizumab plus CT group and 60.2% in the tislelizumab plus CT group, with a statistically significant difference between the two groups (*p* = 0.044). After PSM, the incidence of TRAEs was 51.1% in the pembrolizumab plus CT group and 63.8% in the tislelizumab plus CT group, with no statistically significant difference (*p* = 0.297) (Table 7).

**TABLE 6:** TRAEs in 298 NSCLC patients who received first‐line ICIs therapy.

	Overall (*n* = 298)	< 65 (*n* = 153)	65–69 (*n* = 78)	70–74 (*n* = 48)	≥ 75 (*n* = 19)	*p*
TRAE	158 (53.0)	71 (46.4)	47 (60.3)	29 (60.4)	11 (57.9)	0.135
Fatigue	6 (2.0)	1 (0.7)	1 (1.3)	2 (4.2)	2 (10.5)	0.027
Rash	26 (8.7)	12 (7.8)	6 (7.7)	6 (12.5)	2 (10.5)	0.755
Hypoadrenal function	8 (2.7)	2 (1.3)	4 (5.1)	1 (2.1)	1 (5.3)	0.223
Myocarditis	3 (1.0)	1 (0.7)	0 (0.0)	1 (2.1)	1 (5.3)	0.119
Colitis	4 (1.3)	2 (1.3)	1 (1.3)	1 (2.1)	0 (0.0)	0.858
Myelosuppression	84 (28.2)	34 (22.2)	26 (33.3)	21 (43.8)	3 (15.8)	0.012
Anorexia	3 (1.0)	3 (2.0)	0 (0.0)	0 (0.0)	0 (0.0)	0.792
Diarrhea	3 (1.0)	2 (1.3)	0 (0.0)	1 (2.1)	0 (0.0)	0.527
Nausea	13 (4.4)	8 (5.2)	4 (5.1)	1 (2.1)	0 (0.0)	0.809
Ventosity	2 (0.7)	1 (0.7)	0 (0.0)	1 (2.1)	0 (0.0)	0.468
Hepatitis	14 (4.7)	10 (6.5)	4 (5.1)	0 (0.0)	0 (0.0)	0.270
Myoenzyme abnormality	1 (0.3)	1 (0.7)	0 (0.0)	0 (0.0)	0 (0.0)	1.000
Hypothyroidism	14 (4.7)	6 (3.9)	5 (6.4)	3 (6.2)	0 (0.0)	0.635
Peripheral neuropathy	23 (7.7)	9 (5.9)	12 (15.4)	2 (4.2)	0 (0.0)	0.042
Hemorrhage	3 (1.0)	0 (0.0)	1 (1.3)	0 (0.0)	2 (10.5)	0.005
Immune‐related pneumonitis	12 (4.0)	6 (3.9)	3 (3.8)	3 (6.2)	0 (0.0)	0.789
Radiation pneumonitis	6 (2.0)	3 (2.0)	2 (2.6)	1 (2.1)	0 (0.0)	1.000
Radiation esophagitis	1 (0.3)	0 (0.0)	1 (1.3)	0 (0.0)	0 (0.0)	0.487
Renal injury	3 (1.0)	2 (1.3)	0 (0.0)	1 (2.1)	0 (0.0)	0.527
Hypopituitarism	2 (0.7)	0 (0.0)	2 (2.6)	0 (0.0)	0 (0.0)	0.217

**TABLE 7:** TRAEs mOS of the included population before and after PSM (first‐line use of pembrolizumab + chemotherapy or tislelizumab + chemotherapy).

	Before PSM				After PSM			
	Overall (*n* = 198)	Pembrolizumab + chemotherapy (*n* = 102)	Tislelizumab + chemotherapy (*n* = 96)	*p*	Overall (*n* = 94)	Pembrolizumab + chemotherapy (*n* = 47)	Tislelizumab + chemotherapy (*n* = 47)	*p*
TRAE	104 (52.5)	46 (45.1)	58 (60.4)	0.044	54 (57.4)	24 (51.1)	30 (63.8)	0.297
Fatigue	4 (2.0)	3 (2.9)	1 (1.0)	0.622	2 (2.1)	1 (2.1)	1 (2.1)	1.000
Rash	14 (7.1)	5 (4.9)	9 (9.4)	0.342	5 (5.3)	1 (2.1)	4 (8.5)	0.361
Hypoadrenal function	7 (3.5)	2 (2.0)	5 (5.2)	0.268	5 (5.3)	2 (4.3)	3 (6.4)	1.000
Myocarditis	2 (1.0)	1 (1.0)	1 (1.0)	1.000	2 (2.1)	1 (2.1)	1 (2.1)	1.000
Colitis	3 (1.5)	1 (1.0)	2 (2.1)	0.612	2 (2.1)	1 (2.1)	1 (2.1)	1.000
Myelosuppression	55 (27.8)	22 (21.6)	33 (34.4)	0.064	28 (29.8)	10 (21.3)	18 (38.3)	0.114
Anorexia	3 (1.5)	1 (1.0)	2 (2.1)	0.612	2 (2.1)	0 (0.0)	2 (4.3)	0.495
Diarrhea	3 (1.5)	0 (0.0)	3 (3.1)	0.112	2 (2.1)	0 (0.0)	2 (4.3)	0.495
Nausea	6 (3.0)	3 (2.9)	3 (3.1)	1.000	3 (3.2)	1 (2.1)	2 (4.3)	1.000
Ventosity	1 (0.5)	0 (0.0)	1 (1.0)	0.485	0 (0.0)	0 (0.0)	0 (0.0)	1.000
Hepatitis	6 (3.0)	3 (2.9)	3 (3.1)	1.000	4 (4.3)	3 (6.4)	1 (2.1)	0.617
Hypothyroidism	11 (5.6)	6 (5.9)	5 (5.2)	1.000	7 (7.4)	5 (10.6)	2 (4.3)	0.435
Peripheral neuropathy	18 (9.1)	6 (5.9)	12 (12.5)	0.170	9 (9.6)	3 (6.4)	6 (12.8)	0.486
Hemorrhage	3 (1.5)	3 (2.9)	0 (0.0)	0.247	1 (1.1)	1 (2.1)	0 (0.0)	1.000
Immune‐related pneumonitis	11 (5.6)	6 (5.9)	5 (5.2)	1.000	7 (7.4)	4 (8.5)	3 (6.4)	1.000
Radiation pneumonitis	5 (2.5)	1 (1.0)	4 (4.2)	0.201	3 (3.2)	1 (2.1)	2 (4.3)	1.000
Renal injury	3 (1.5)	1 (1.0)	2 (2.1)	0.612	0 (0.0)	0 (0.0)	0 (0.0)	1.000
Hypopituitarism	2 (1.0)	0 (0.0)	2 (2.1)	0.234	1 (1.1)	0 (0.0)	1 (2.1)	1.000

## 4 Discussion

In this study, no statistically significant differences were observed in the efficacy and safety of ICIs treatment across different age groups. Given that both elderly and younger patients were included in this study, the clinical characteristics grouped by age did not show significant statistical differences, allowing for a direct comparison of the efficacy and safety of ICIs treatment among the age groups. Although there was a significant difference in the number of patients who received radiotherapy across age groups, with more patients in the < 65 years old group receiving radiotherapy and fewer in the elderly group, no significant difference was found in the mOS among the different age groups. This suggests that radiotherapy may not offer survival benefits for elderly patients with advanced NSCLC. Currently, only a few studies have used real‐world data to directly compare the response to first‐line ICIs treatment between elderly and young patients. A pooled analysis from Nosaki et al., based on the Keynote‐010, Keynote‐024, and Keynote‐042 trials, indicated that patients aged 75 and older still derived survival benefits from pembrolizumab treatment compared to those under 75 years of age [14].

A phase II prospective study conducted in Japan showed a median overall survival of 21.6 months for pembrolizumab‐treated patients aged 70 years and older with advanced NSCLC, suggesting that people aged 70 years and older can still benefit from ICIs [39]. However, subgroup analyses of RCT studies such as IMpower150, CheckMate‐227, and CheckMate 9LA suggest that while patients aged 65–74 years still benefit from immunotherapy in terms of survival compared to patients under 65, while patients aged over 75 may not benefit [15, 25, 40]. A meta‐analysis involving 10,291 NSCLC patients indicated that ICIs prolonged the mOS of NSCLC patients in the aged < 65‐year‐old group and the 65–74‐year‐old group. However, in the ≥ 75‐year‐old group, ICIs did not prolong the mOS of NSCLC patients [41]. These results and our study suggested that age alone should not be considered a barrier for elderly patients to receive ICIs.

In this study, patients with advanced NSCLC and PD‐L1 TPS ≥ 50% had greater benefits from ICI treatment compared to those with PD‐L1 TPS < 1% or PD‐L1 TPS1‐ 49%. The mOS was significantly longer in the PD‐L1 TPS ≥ 50% group. This finding is consistent with many previously known research results. For instance, the Keynote‐189 study demonstrated that the 1‐year survival rate of patients with PD‐L1 TPS ≥ 50% who received pembrolizumab plus Tchemotherapy was 73%, with a 52% reduction in mortality risk. Meanwhile, the 1‐year survival rates of PD‐L1 < 1% or those with PD‐L1 TPS 1‐49% were 61.7% and 71.5%, respectively [22, 42, 43, 44]. PD‐L1 is currently one of the most commonly used predictive biomarkers for assessing the efficacy of ICIs in clinical practice. However, due to intratumoral heterogeneity and variability among different PD ‐ L1 antibodies, PD‐L1 scoring results can vary significantly, therefore show certain limitations in its predictive reliability. In our study, we further explored the related factors of hematological biomarkers for predicting the efficacy of ICIs in patients with advanced NSCLC. Currently, potential predictive biomarkers such as NLR, PLR, SII, and PNI have been reported in the literature [36, 38, 45, 46, 47]. However, the cutoff values for NLR and PLR differ across studies, and optimal cutoff value has yet been established for these hematological indicators. Given that blood tests are easily accessible and cost‐effective, hematological markers represent a promising direction. We look forward to future prospective studies to further clarify the relationship between hematological biomarkers and ICI efficacy in NSCLC.

Since 2015, several landmark trials, including Keynote‐010, Keynote‐024, Keynote‐042, Keynote‐189, and Keynote‐407, have confirmed the pivotal role of pembrolizumab combined with chemotherapy in the treatment of advanced NSCLC, significantly prolonging patient survival [13, 21, 22, 43, 48]. Multiple RCTs have also demonstrated that the Chinese‐developed tislelizumab can improve the prognosis of patients with advanced NSCLC [23, 24, 49]. A retrospective study involving 164 patients with advanced lung squamous cell carcinoma in China showed that first‐line treatment with sintilimab plus CT and pembrolizumab plus CT resulted in ORR of 61.4% and 65.1%, respectively, with a (DCR) of 92.1% in both groups. The mOS was NA in the pembrolizumab group and 30.7 months in the sintilimab group (HR 1.045, 95% CI: 0.607–1.802), indicating similar efficacy between the two treatments [50]. Our study is the first retrospective analysis directly comparing the efficacy and safety of pembrolizumab plus CT versus tislelizumab plus CT in patients with advanced NSCLC. Our results suggest that the efficacy of the two regimens is broadly comparable. In terms of safety, before PSM, the incidence of TRAEs was slightly higher in the tislelizumab group compared to the pembrolizumab group. However, after PSM, no significant difference in safety was observed between the two groups.

However, this study still has several limitations. First, as a retrospective study, we were unable to collect precise information for most patients, such as the time to disease progression and daily activity tolerance. As a result, we did not assess the relationship between mPFS in different age groups. Secondly, all patients included in our study had good PS 0–1; patients with poorer physical condition (PS 2–3) were not enrolled. Therefore, the findings cannot be generalized to patients with poorer physical condition. Third, the sample size for the comparison between pembrolizumab plus CT and tislelizumab plus CT was relatively small, which may influenced the robustness of the results. In conclusion, age should not be considered a barrier for patients with advanced NSCLC to receive ICIs treatment. Instead, a comprehensive clinical assessment is essential to guide treatment decisions. PD‐L1 expression remains a valuable predictive biomarker, while hematological indicators such as NLR and PNI may serve as prognostic markers for first‐line ICIs therapy in NSCLC. Our findings suggested that the efficacy and safety profiles of pembrolizumab plus CT and tislelizumab plus CT show no differences in the treatment of advanced NSCLC. Future randomized controlled trials focusing on first‐line ICIs treatment in elderly patients with advanced NSCLC were warranted to further validate these results.

## Author Contributions

Sheng Yang collected, analyzed the data, and drafted the manuscript. Yalin Zhu and Sitong Liu collected the data. Sheng Yang and Kui Xiao revised the manuscript. All authors contributed to the article and approved the submitted version.

## Funding

This work was supported by the Natural Science Foundation of Hunan Province (2021JJ30963), Scientific Research Project of Hunan Provincial Health Commission (202103020704), and the National Key Clinical Specialty Construction Projects of China.

## Ethics Statement

This retrospective study has been approved by the Hospital Ethics Committee of the Second Xiangya Hospital of Central South University (LYF2024299), and an application for informed consent exemption has been made. Throughout the entire process of data collection, storage, and sharing, measures have been taken to ensure the confidentiality of participants' data.

## Conflicts of Interest

The authors declare no conflicts of interest.
